# Muse cells and induced pluripotent stem cell: implication of the elite model

**DOI:** 10.1007/s00018-012-0994-5

**Published:** 2012-04-24

**Authors:** Masaaki Kitada, Shohei Wakao, Mari Dezawa

**Affiliations:** Department of Stem Cell Biology and Histology, Tohoku University Graduate School of Medicine, Sendai, Miyagi 980-8575 Japan

**Keywords:** Stochastic model, Elite model, Tumorigenicity, Adult stem cells, Mesenchymal stem cells

## Abstract

Induced pluripotent stem (iPS) cells have attracted a great deal attention as a new pluripotent stem cell type that can be generated from somatic cells, such as fibroblasts, by introducing the transcription factors Oct3/4, Sox2, Klf4, and c-Myc. The mechanism of generation, however, is not fully understood. Two mechanistic theories have been proposed; the stochastic model purports that every cell type has the potential to be reprogrammed to become an iPS cell and the elite model proposes that iPS cell generation occurs only from a subset of cells. Some reports have provided theoretical support for the stochastic model, but a recent publication demonstrated findings that support the elite model, and thus the mechanism of iPS cell generation remains under debate. To enhance our understanding of iPS cells, it is necessary to clarify the properties of the original cell source, i.e., the components of the original populations and the potential of each population to become iPS cells. In this review, we discuss the two theories and their implications in iPS cell research.

## Introduction

In 2006, artificially-induced pluripotent stem (iPS) cells were reportedly generated from mouse fibroblasts by introducing exogenous Oct3/4, Sox2, Klf4, and c-Myc (the so-called Yamanaka factors) [1]. These cells, named iPS cells, have attracted much attention as a new stem cell type with potential for medical research and clinical applications. Although several studies have evaluated the potential use of human embryonic stem (ES) cells in cell-based therapy, ethical concerns relating to the use of cells obtained from embryos limit their practical application. Thus, iPS cells, which can be generated from somatic cells, are expected to resolve the problems that pertain to ES cells [2]. Furthermore, iPS cells from patients with intractable disease could provide a valuable system for analyzing the mechanism of disease onset in vitro. Drug screening using iPS cells is also conceivable. The use of human ES cells has been limited to certain established clones, and thus immunologic rejection is considered a major obstacle for cell therapy, whereas patient-derived iPS cells would be theoretically free from immunorejection.

The basic characteristics of iPS cells are similar to those of ES cells; they express pluripotency markers, show self-renewal, and differentiate into cells representative of all three germ layers. Like ES cells, iPS cells show unlimited proliferative activity and form teratomas upon transplantation [3].

Ongoing research, however, has revealed differences between iPS and ES cells with respect to epigenetic modification, heterogeneity, and differentiation potential. For example:

- iPS cells exhibit distinct epigenetic differences from ES cells that are caused by aberrant methylation during early passages [4].
- iPS cells harbor residual DNA methylation signatures, namely “epigenetic memory”, characteristic of their somatic tissue of origin, which favors their differentiation along lineages related to the donor cell, while restricting alternative cell fates [5–7].
- iPS cells obtained from mouse fibroblasts, hematopoietic, and myogenic cells exhibit distinct transcriptional and epigenetic patterns. Their cellular origin influences in vitro differentiation potential, and continuous passaging of iPS cells largely attenuates these differences [4].
- The blood-forming potential of iPS cells derived from early bone marrow cells is higher than that of iPS cells derived from neural progenitor cells, whereas the potential is the same between nuclear transfer-ES cells and fertilized embryo-derived ES cells [8].
- The same tendency is observed for blood and keratinocyte derivatives. As a consequence of the incomplete erasure of tissue-specific methylation and aberrant de novo methylation, umbilical cord blood-derived and neonatal keratinocyte-derived iPS cells are distinct in their genome-wide DNA methylation profiles and differentiation potential; umbilical cord blood-derived cells have higher potential to differentiate into hematopoietic lineage cells, and neonatal keratinocyte-derived iPS cells have higher potential to differentiate into keratinocytes [9].
- Epigenetic abnormalities that arise during early reprogramming are inherited by iPS cells. These include hundreds of abnormal gene silencing events, patterns of aberrant responses to epigenetic-modifying drugs resembling those of cancer cells, and the presence of cancer-specific gene promoter DNA methylation alterations [10].
- With regard to a theoretical benefit of immune-tolerance in iPS cells derived from autologous cells, a recent report demonstrated that, in contrast to ES cell derivatives, abnormal gene expression in some cells differentiated from iPS cells can induce T cell-dependent immune responses in syngeneic recipients [11].

Such characteristics of iPS cells raise a number of questions. What is the mechanism that underlies the generation of iPS cells? Why do iPS cells drag epigenetic memory? How are tumorigenic properties conferred on iPS cells concomitant with pluripotency? Why is the generation ratio still very low? Perhaps these questions have their origin in one more basic question: what is the entity of iPS cells? This question will be answered by elucidating the generation mechanism.

To date, two mechanistic theories of iPS cell generation, the stochastic and the elite models, have been proposed [12]. The stochastic model purports that every cell type can potentially be reprogrammed to become an iPS cell by introducing Oct3/4, Sox2, Klf4, c-Myc, Nanog, and Lin28 [2, 13]; and the elite model proposes that iPS cells can be generated from only subsets of cells [12]. The correct model, however, remains an open question, and both models are conceivable. In any case, the mechanism of iPS cell generation is still veiled in mystery. At present, the focus of iPS cell research has moved from advancing their efficiency to evaluating it in each disease model aiming for application to cell-based therapy. Before proceeding, however, the fundamental questions of what iPS cells are and how are they generated must be addressed. Without this basic understanding, iPS cell research cannot advance. This review focuses on the generation of iPS cells and discusses the entity of iPS cells.

## The stochastic model of iPS cell generation

The stochastic model is now broadly accepted. iPS cells have been generated from various cell sources, such as skin fibroblasts [2]; keratinocytes [14]; mesenchymal cells from fat tissue [15], oral mucosa [16] and dental pulp [17]; cord blood cells [18]; and peripheral blood cells [19] in humans; and are therefore considered to be generated from any cell types. Likewise, T cells are reported to be a source for iPS cells so that even differentiated peripheral blood cells can be reprogrammed to iPS cells [20]. Jaenisch and colleagues argued that the existence of distinct cell division rate-dependent and -independent modes accelerates the stochastic course of reprogramming and that the number of cell divisions is a key parameter driving epigenetic reprogramming to pluripotency, and thus that, theoretically, almost all mouse donor cells eventually give rise to iPS cells with continued growth and transcription factor expression [13]. Other investigators have focused on epigenetic regulation after establishing iPS cells. Nishino et al. [21] reported that stochastic de novo methylation of genomic DNA occurs, and that cell division proceeds in established iPS cells after prolonged culture, leading to a cell condition that epigenetically more closely resembles that observed in ES cells, suggesting that iPS cell generation is regulated by such stochastic epigenetic events. While these reports theoretically and logically support the stochastic model of iPS cell generation, rigorous proof that all cell types including fully differentiated cells are, in a strict sense, able to become iPS cells is still awaited.

## Mesenchymal stem cells (MSCs) as a source of iPS cells: their heterogeneity and diversity

Fibroblasts are the most popular original cell source for generating iPS cells [1, 2]. They are usually collected from adherent dermal cell cultures. Histologically, however, the dermis comprises various cell types; although fibroblasts are the major component of the connective tissue, blood vessel-associated cells such as endothelial cells and pericytes are also at least present in the dermis. Furthermore, the adult dermis contains several types of stem or progenitor cells, such as skin-derived precursors, neural crest-derived stem cells, melanoblasts, perivascular cells, endothelial progenitors, and adipose-derived stem cells [22–29]. Therefore, while cells cultured from the dermis mainly contain authentic fibroblasts, many other cell types are included. In fact, primary cultured dermal cells subjected to subculture contain cells positive for CD117 (a marker for melanoblasts), CD146 (perivascular cells and adipose-derived stem cells), CD271 (neural crest-derived stem cells), Snai1 (skin-derived precursors), and Slug (skin-derived precursors) [30]. Thus, dermal fibroblasts are actually not a single cell type, but rather comprise heterogeneous cell populations.

This is the same for another mesenchymal tissue, bone marrow. Bone marrow-derived mesenchymal cells (often called bone marrow mesenchymal stem cells; BM-MSCs) are usually collected as adherent cells from bone marrow aspirates and are also heterogeneous. Pittenger et al. [31] were the first to analyze the cell surface antigens of BM-MSCs in detail. Like human fibroblasts, BM-MSCs are uniformly positive for SH2, SH3, CD29, CD44, CD71, CD90, CD106, CD120a, CD124, and many other surface antigens, but negative for markers of the hematopoietic lineage, including a monocyte antigen CD14, a hematopoietic progenitor cell antigen CD34, and the leukocyte common antigen CD45 [31, 32].

Like fibroblasts and BM-MSCs, MSCs are generally a crude cell population because they are usually harvested as adherent cells from mesenchymal tissues such as the dermis, bone marrow, adipose tissue, and umbilical cord. Overall, MSCs express mesenchymal markers, but detailed analyses reveal that the marker content and expression ratios differ among these cells. Therefore, it must be kept in mind that mesenchymal cells, even commonly used fibroblasts, often differ with regard to their origin, phenotype, and differentiation state. As a consequence, when MSCs are targeted for iPS cell generation, the basic cell population is heterogeneous in the potential to become iPS cells.

When culturing cells from other organs and tissues other than mesenchymal tissues (e.g., peripheral nerve, muscle, liver, and kidney), fibroblasts are easily mixed into the primary culture. Even in immune systems such as the spleen, primary cultured cells are not free from fibroblasts. In other words, contamination of mesenchymal cells is unavoidable and collection of a single population is not guaranteed unless the cells are strictly labeled by cell surface markers and collected by cell sorting. Further, histologically, almost all organs contain connective tissue, and therefore mesenchymal cells will easily penetrate into the primary culture from any organ harvested. It is not surprising that even peripheral blood is not free from mesenchymal cells because several studies have demonstrated that MSCs with multilineage differentiation ability appear in the blood under many circumstances such as disease or injury [33–37].

Hochedlinger’s group suggested that the differentiation stage of the starting cell influences the efficiency of reprogramming into iPS cells [4]. They tested the potential of mouse hematopoietic cells at different stages of differentiation to be reprogrammed into iPS cells and demonstrated that hematopoietic stem and progenitor cells give rise to iPS cells with much higher efficiency than do terminally differentiated B cells. Another report suggested that many adult tissues contain tissue stem cells that already express pluripotency markers such as Oct3/4, and that those cells contribute to iPS cell generation [38, 39]. As these papers suggest, cells in an undifferentiated state are better able to generate iPS cells.

A problem in the current iPS cell research is that in most cases experiments are conducted using a mixture of cells with different stages, potential, and origin. The generation ratio of iPS cells is still very low, and only a small number of cells develop into iPS cells. In such circumstances, the signal coming from cells truly attempting to become iPS cells will be drowned out by the noise of background cells, making it difficult to unveil the actual mechanism of iPS cell generation.

There are some reports that iPS cells are successfully generated by reprogramming terminally differentiated cells. Although iPS cells appear to be generated from terminally differentiated cells from various organs such as the liver [40], spleen [41], or peripheral blood [20], these results may not, in a strict sense, rule out the possibility that iPS cells are generated from cells other than terminally differentiated cells unless those terminally differentiated cells are strictly identified and selected, e.g., using FACS, before subjecting the cells to the iPS cell-generation procedure.

## Definition of pluripotent stem cells

A “pluripotent” cell is defined as that having the ability to give rise to cell types of all three embryonic germ layers, namely endodermal, mesodermal, and ectodermal cells [42]. In the case of “pluripotent stem cells”, the concept “stem cell” applies not only to the differentiation potential but also the ability to self-renew. In many cases, pluripotent stem cells show germline transmission and/or teratoma formation in addition to the above two requirements, mimicking normal development [42, 43]. Epiblast stem cells, however, a type of pluripotent stem cell, do not form teratomas under certain circumstances [44]. Therefore, pluripotent stem cells do not always meet the strict requirements of teratoma formation or germline transmission.

On the other hand, MSCs differentiate into a broad spectrum of cells that crosses the oligolineage boundaries between mesodermal and ectodermal or endodermal lineages [45]. Some of the cell types that belong to mesenchymal tissues, such as neural crest-derived stem cells and skin-derived precursors, show diploblastic differentiation (mesodermal- and ectodermal-lineage cells), and such differentiation ability is called ‘multipotency’ [23, 28]. Although there are a few reports demonstrating that a subpopulation of MSCs generate cells representative of all three germ layers, the term multipotency is not adequate to describe the high differentiation ability of these cells. In fact, such cells are often called ‘pluripotent’ to describe their high differentiation ability [46–49]. In summary, the abilities of self-renewal and differentiation into cells representative of all three germ layers are essential and common requirements for pluripotent stem cells, and these two properties are sufficiently comprehensive to represent their high differentiation ability rather than setting limits by including germline transmission and/or teratoma formation abilities. Therefore, in this review, we define “pluripotent stem cells” as cells having the ability to self-renew and to differentiate into cells representative of all three germ layers.

## Mesenchymal cells contain pluripotent stem cells

In general, tissue stem cells generate the cell types of the tissue in which they reside, and thus the range of their differentiation capabilities is usually limited. For example, hematopoietic stem cells generate blood cells and neural stem cells generate neurons and glial cells [50–52]. MSCs differ from other tissue stem cells in that they differentiate not only into the same mesodermal-lineage, such as bone, cartilage, and adipocytes, but also into other lineages, ectodermal and endodermal cells.

When MSCs are treated with a certain sets of cytokines or with transient gene introduction, they differentiate in vitro into cell types including endothelial cells [53], cardiac muscle [54], skeletal muscle [55], hepatocytes [56], neuronal cells [57], peripheral glial cells [58], insulin-producing cells [59], and epithelial cells [60]. The broad spectrum of differentiation observed in MSCs does not occur in a high ratio, and thus the cells responsible for differentiation were considered to comprise a subpopulation of MSCs. Differentiation of MSCs into hepatocytes [61], keratinocytes [37], and cardiac muscles [62] is also recognized in vivo in disease models, albeit with a very low frequency*.* These observations lead us to speculate that MSCs contain a subpopulation of pluripotent cells.

**Fig. 1:**
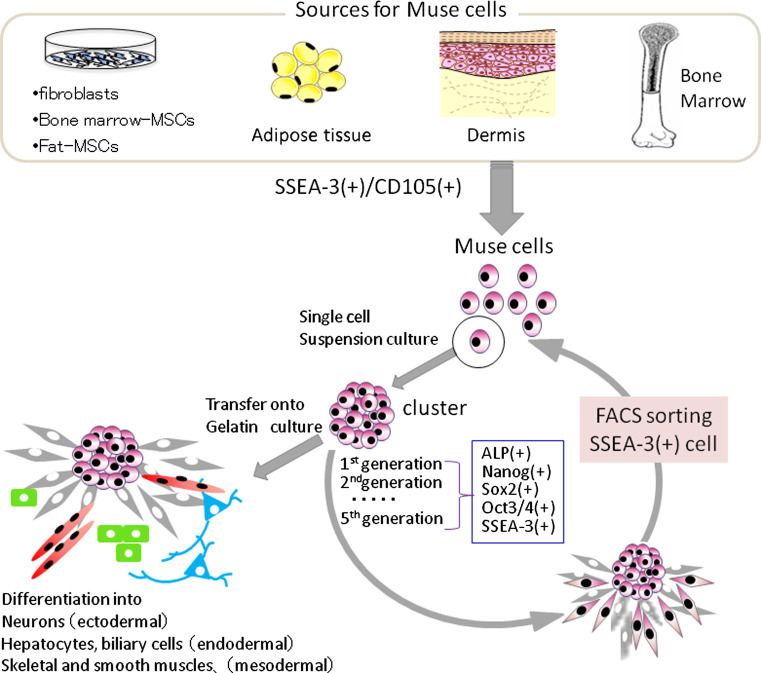
Properties of Muse cells. Muse cells can be collected from cultured mesenchymal cells (fibroblasts, bone marrow-MSCs, or fat-MSCs) and mesenchymal tissues (adipose tissue, dermis, and bone marrow aspirates) as cells double-positive for SSEA-3 and CD105. After isolating Muse cells by FACS, single Muse cells cultured in suspension (single cell suspension culture) generate characteristic clusters that express markers related to pluripotency [alkaline phosphatase (ALP), Nanog, Sox2, Oct3/4, SSEA-3]. When cell clusters were transferred onto gelatin culture and spontaneous differentiation was induced, cells with endodermal- (alpha-fetoprotein + cells), ectodermal- (neurofilament + cells), and mesodermal- (desmin + cells) lineage were observed. We confirmed that Muse cells continued to self-renew up to the fifth generation, indicating that they are pluripotent

Recently, adult human mesenchymal cells such as BM-MSCs and dermal fibroblasts were shown to contain pluripotent stem cells that were named multilineage-differentiating stress-enduring (Muse) cells [32]. These cells can be isolated as cells that are double-positive for the pluripotency marker stage-specific embryonic antigen-3 (SSEA-3, a marker for undifferentiated human ES cells) and for a mesenchymal marker CD105. When a single Muse cell was cultured in suspension, the cell began to proliferate and form a cell cluster resembling an embryoid body of ES cells. The cluster expressed the pluripotency markers SSEA-3, Nanog, Oct3/4, and Sox2 and was positive for alkaline phosphatase, and cells in the cluster differentiated into endodermal-, ectodermal-, and mesodermal-lineage cells when cultured on the gelatin-coated dish [32] (Fig. 1).

Although the existence of pluripotent cells in MSCs has long been suggested, to date there have been no reports clearly demonstrating self-renewal and differentiation potency at a single cell level, so that the pluripotency in MSCs has remained controversial [63, 64]. Most importantly, single Muse cells are able to generate cells representative of all three germ layers: mesodermal-lineage (osteocytes, adipocytes, chondrocytes, skeletal muscle cells, smooth muscle cells), ectodermal-lineage (neuronal cells, glial cells, epidermal cells), and endodermal-lineage (hepatocytes, biliary system cells), and they self-renew for up to five generations; thus, they are pluripotent stem cells [32] (Fig. 1).

ES cells and iPS cells are pluripotent stem cells that form teratomas upon transplantation. It is noteworthy that, in contrast to these pluripotent stem cells, Muse cells do not undergo tumorigenic proliferation, and do not develop into teratomas when transplanted into immunodeficient mouse testes [32]. Consistently, while ES cells and iPS cells have high telomerase activity, Muse cells have low telomerase activity similar to somatic cells such as fibroblasts. Genes related to cell-cycle progression are extensively upregulated in human ES and iPS cells, but in Muse cells they are expressed at the same level as in naive fibroblasts [30]. The non-tumorigenicity of Muse cells seems to be consistent with the fact that they reside in normal adult mesenchymal tissue.

The ratio of Muse cells is <1 % in cultured BM-MSCs and 2–5 % in commercially obtained fibroblasts, but it is very low in the fresh human bone marrow mononucleated cell fraction (1 of 3,000 mononucleated cells) [32]. Immunohistochemistry experiments demonstrated that Muse cells locate sparsely in the connective tissues of organs and do not associate with any particular structure such as blood vessels [30].

## The elite mechanistic model of iPS cell generation

In parallel with the stochastic model, it is argued that iPS cells are the result of the procurement of tumorigenic proliferative activity in adult stem cells [65–69]. This, however, has not been fully investigated. Byrne et al. [67] reported that only SSEA-3-positive human dermal fibroblasts cells can generate iPS cells, but the characteristics of the original SSEA-3-positive cells were not fully evaluated. Therefore, the process of iPS cell generation from this cell population remains obscure, particularly with regard to whether these cells acquired the abilities of self-renewal and differentiation into cells representative of all three germ layers only after transduction of the four Yamanaka factors or whether they originally possessed these abilities.

**Fig. 2:**
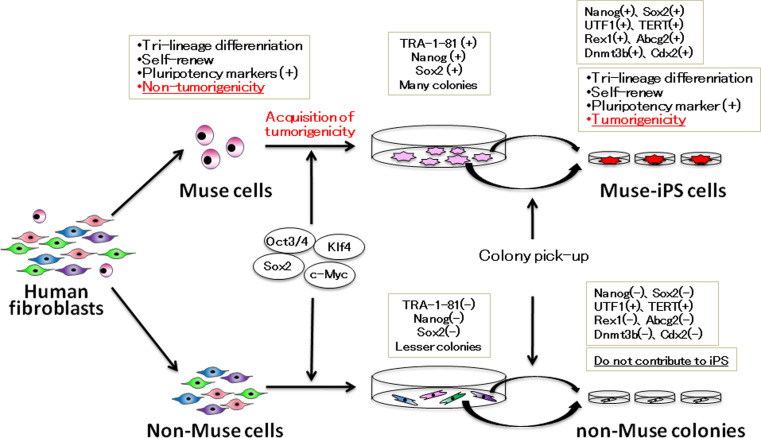
When human fibroblasts were separated into Muse and non-Muse cells and each population subjected to iPS cell generation, iPS cells are generated only from Muse cells and never from non-Muse cells. The properties of each cell population are shown in the *boxes*

A recent report suggested that, at least in the case of human fibroblasts, iPS cells are generated only from pluripotent Muse cells, which supports the elite model [30]. As mentioned, Muse cells reside in human mesenchymal tissues and mesenchymal culture cells and exhibit the characteristic properties of pluripotent stem cells, although they do not show tumorigenic properties. Interestingly, when Muse cells were removed from human dermal fibroblasts, the remaining cell population was unresponsive to the Yamanaka factors and failed to generate iPS cells [30]. When human fibroblasts were separated into Muse cells and non-Muse cells, and each population was subjected to the iPS cell generation procedure, iPS colonies were only generated from Muse cells and not from non-Muse cells. Just prior to colony pickup, both populations formed colonies with various morphologic features, but only the Muse cell population produced colonies with a human ES cell-like morphology that were positive for the human pluripotent stem cell marker TRA-1–81, a marker for promising iPS colonies [70], while non-Muse cells generated no TRA-1–81-positive colonies and all the colonies from non-Muse cells were unlike human ES cells. All the cells and colonies of each population were collected and subjected to reverse transcription-polymerase chain reaction (RT-PCR), which detected endogenous Sox2 and Nanog, the fundamental transcriptional regulators of pluripotent stem cells in cells and colonies derived from Muse cells, but never in those derived from non-Muse cells [30] (Fig. 2). Colonies generated from Muse and non-Muse cells were further picked up and passaged in individual wells to establish iPS cell lines. Only colonies picked from Muse cells established iPS cells (Muse-iPS cells), and colonies originating from non-Muse cells (non-Muse colonies) were unlike human ES or iPS cells in their morphology and failed to establish iPS cells. iPS cells-derived from Muse cells expressed not only Oct3/4, Sox2, and Nanog but also Rex1, UTF1, TERT, Abcg2, Dnmt3b, and Cdx2. These cells differentiated into endodermal-, ectodermal-, and mesodermal-lineage cells in vitro, and formed teratomas after injection into immunodeficient mouse testes [30].

It is easy to understand that Muse cells that already show pluripotency are more susceptible than non-Muse cells to becoming iPS cells, but the more important question is why none of the non-Muse cells developed into iPS cells. Indeed, non-Muse cell-derived colonies did not express the fundamental transcriptional regulators for pluripotent stem cells, such as endogenous Oct3/4, endogenous Sox2, or Nanog, but nor did they express Rex1, Abcg2, Dnmt3b, or Cdx2, which have been known to indicate the reprogramming state of colonies [71]. Chan et al. [71] reported that colonies generated during iPS cell generation can be divided into type I, II, and III colonies: type I colonies, which do not express Rex1, Abcg2, Dnmt3b, and Cdx2, do not develop into iPS cells and but remain in the incompletely reprogrammed state; type II colonies, which do not express Rex1, Abcg2, or Dnmt3b, but do express Cdx2, occasionally spontaneously transit to iPS cells; and type III colonies, which express these four genes and are identified as iPS cell colonies. In this context, non-Muse cell-derived colonies negative for Rex1, Abcg2, Dnmt3b, and Cdx2 correspond to type I colonies that stay arrested at an early stage of iPS cell generation and thus do not develop into iPS cells [30] (Fig. 2).

The inability of non-Muse cells to respond to the Yamanaka factors could also be seen in the methylation state of the promoter regions of Nanog and Oct3/4. In the naive state, the Nanog and Oct3/4 promoter regions are more methylated in non-Muse cells than in Muse cells. In Muse cells, however, those partly methylated promoter regions become completely demethylated when they develop into iPS cells. On the other hand, such demethylation of the promoter regions of Nanog and Oct3/4 is never observed in non-Muse cell-derived colonies [30]. Those phenomena were all repeated using a single polycistronic Oct3/4–Klf4–Sox2–c-Myc–GFP-expressing viral vector encoding all four factors, confirming that all of the above phenomena are not caused by unsuccessful transduction of one or more of the four retroviral vectors encoding Oct3/4, Sox2, Klf4, and c-Myc [30].

**Fig. 3:**
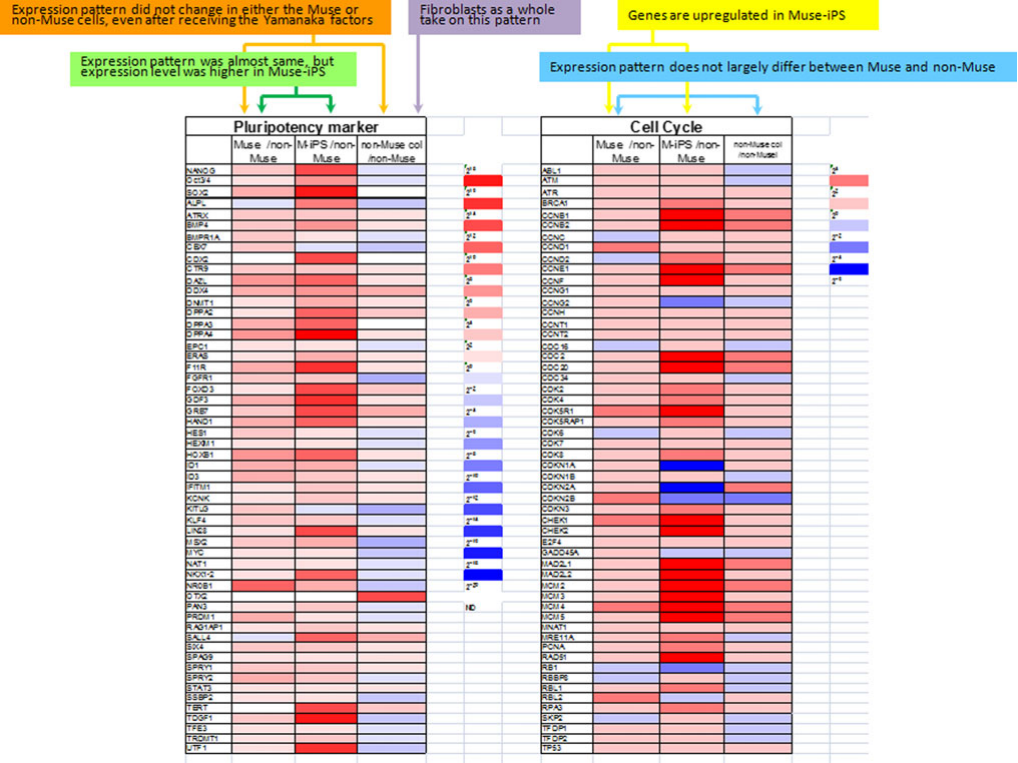
Gene expression pattern in *Muse*, Muse-iPS (*M-iPS*), *non-Muse*, and non-Muse colonies (*non-Muse col*). The expression pattern of pluripotency markers in Muse cells and Muse-iPS was almost the same, but expression level was higher in Muse-iPS cells than in naive Muse cells (*green*). Neither Muse nor non-Muse cells showed a change in the expression pattern of pluripotency markers even after receiving the Yamanaka factors (*orange*). While naïve fibroblasts are known to contain Muse cells, the expression pattern and level of pluripotency markers in the fibroblasts as a whole takes on the pattern of non-Muse cells (*purple*). Genes related to cell cycle progression did not largely differ between Muse and non-Muse cells (*blue*), but they were upregulated when Muse cells became Muse-iPS cells (*yellow*). (Modified version of table 1 in Ref. [30])

Gene expression profiles provide information about cell responsiveness to the Yamanaka factors. As for genes related to pluripotency, the “expression level” is lower in naive Muse cells than in Muse-iPS cells, but the “expression pattern”, namely the repertoire of genes expressed, is nearly the same between naive Muse cells and Muse-iPS cells. In contrast, naive non-Muse cells do not express genes related to pluripotency, and neither the expression level nor pattern show substantial changes even after receiving the Yamanaka factors, namely in non-Muse colonies (Fig. 3). Genes related to cell-cycle progression were mostly upregulated in Muse cell-derived iPS cells as compared with naive Muse cells. This is consistent with the fact that naive Muse cells have lower telomerase activity and do not form teratomas after transplantation into immunodeficient mouse testes, while Muse-iPS cells formed teratomas. In non-Muse cell-derived colonies, some of the genes related to cell-cycle progression were upregulated compared with those in naive non-Muse cells, but the upregulation was marginal and not as extensive as in Muse-iPS cells [30] (Fig. 3).

## What kind of ability does the Yamanaka factors confer on the cells?

The most noteworthy observation of these gene expression patterns is that, regardless of whether the cells are Muse or non-Muse cells, the expression pattern of genes related to pluripotency is not altered by introduction of the Yamanaka factors (Fig. 3). In other words, introduction of the Yamanaka factors does not alter the cell state in terms of its differentiation ability. Although Muse cells express the pluripotency-related genes, it is reasonable that the gene expression profile in adult human dermal fibroblasts will be as same as that in non-Muse cells because the ratio of Muse cells in dermal fibroblasts is only several percent [30], so that the signal from Muse cells is masked by the vast majority of non-Muse cells (Fig. 3). When the pluripotency gene expression pattern of fibroblasts changed to that of iPS cells, then it seems that introducing the Yamanaka factors brought terminally differentiated cells back to the cell state resembling that of the inner cell mass cells. The differences in the results of Muse and non-Muse cell experiments clearly indicate that this did not happen in human fibroblasts.

Apart from these issues, the question of how Muse cells become iPS cells remains to be clarified. Muse cells are originally non-tumorigenic, but when they become iPS cells, they newly acquire tumorigenic proliferation activity while retaining their pluripotency. It is noteworthy that Nanog and Oct-4 accelerate cell-cycle progression in pluripotent stem cells such as ES cells [72, 73]. It is also reported that over-expression of Oct4 caused hyperplasia in the new-born mice [74]. Thus, it is possible that the generation of iPS cells from Muse cells requires a much higher expression of critical transcription factors including pluripotency markers that may lead to the activation of genes related to cell-cycle progression, which is followed by further increases in the pluripotency marker expression levels. Such synergistic effects may result in higher expression levels of genes related to pluripotency as well as to cell-cycle progression in Muse cell-derived iPS. The characters of Muse cells in terms of homogeneity and their derivation from different mesenchymal sources (such as skin and bone marrow) have not been fully elucidated, so that the responsiveness of each Muse cell to the Yamanaka factors should be clarified as a future issue.

In the framework of Muse and non-Muse cells, human fibroblasts can be divided into two populations: cells that primarily contribute to iPS cell generation and those that do not. These results demonstrate that the human fibroblast system fits into the elite model of iPS cell generation. Further studies will clarify the potential of this system to generate iPS cells from other tissues and cell types.

## The necessity for unified criteria to identify iPS cells

Initially, iPS cells were reported to be generated from mice and human fibroblasts with very low efficiency, nearly 0.001 %, [1, 2], but many recent attempts have been made to improve the generation efficiency. For example, combining gene introduction with the use of reagents such as valproic acid, or inhibitors for TGF-beta, MAPK/ERK, or suppression of p53 is reported to increase the efficiency of iPS cell generation [75–77]. More recently, a replication-defective and persistent Sendai virus vector containing *Oct4*/*Sox2*/*Klf4*/*c*-*Myc* induced iPS cell from mouse primary fibroblasts with an efficiency of ~1 %, as estimated by green fluorescent protein expression driven by the Nanog promoter [78]. Similarly, replacing c-Myc with Glis1 increased iPS cell generation from human fibroblasts with an efficiency of ~0.16 %, also based on Nanog promoter activity [79]. As for the use of valproic acid, the efficiency in mouse embryonic fibroblasts was increased up to ~2–3 %, based on Oct4-green fluorescent protein quantification [80]. Despite these efforts, however, the generation efficiency is still far from being very high. Even in the case of Muse cells, generation efficiency is only 0.03 %, albeit counted strictly based on the expression of Nanog, endogenous Oct3/4, and Sox2 as well as Rex1, Abcg2, Dnmt3b, and Cdx2. This efficiency corresponds to 30 times greater efficiency than naive fibroblasts [30].

As evidenced by these reports, the primary problem in iPS cell research is that the criteria for iPS cell generation differs among reports; some reports calculate generation efficiency based only on ALP staining, whereas others base generation efficiency on the expression of a single pluripotency marker. Because of the current lack of unified criteria to identify the generation of iPS cells, the reported generation efficiencies cannot be compared with each other. In fact, not all colonies positive for ALP staining are iPS cells, and likewise, not all colonies that are positive for the expression of a reporter gene product driven by only by a single pluripotency-related gene promoter such as Nanog or Oct3/4 meet the strict criteria for iPS cells [70, 81]. Previously, gene expression analyses in live images and quantitative PCR were performed both in colonies resembling and colonies not resembling ES cells and revealed that the expression of Nanog or Oct3/4, or positive reaction for ALP, occur in various kinds of colonies other than iPS cells, and thus suggest that both factors are unreliable for the identification of iPS cells [71, 82]. In addition, tissue stem cells are occasionally positive for Oct3/4- or Nanog, implying that a single marker expression of these genes will also not indicate the cells in the pluripotent state [38, 39, 66]. These findings indicate that the calculation of iPS cell generation based on the single expression of Oct3/4 or Nanog will likely overestimate the number of iPS cells. Unified and reliable criteria to identify iPS cells must be firmly established.

## Perspectives

Many reports have focused on the interpretation of the output of iPS cell generation, but understanding the properties of the original starting cell population for generating iPS cells is important for understanding their generation mechanism. Indeed, when the emergence of iPS cells is unforeseeable, it seems that all cells have the potential to become iPS cells and that iPS cells are stochastically generated by coincidence combined with an exquisite balance of intrinsic factors. On the other hand, pluripotent cells such as Muse cells are recognized among the original cell population, and iPS cells are exclusively generated from these cells; thus, we now recognize that the stochastic model is not the only viable theory of iPS cell generation. Therefore, we must turn our attention to the heterogeneity and diversity of the original cell population. The major publication regarding the mechanism of iPS cell generation and characterization is summarized in Table 1.

**Table 1:** Summary of published articles that relate to the mechanism of iPS cell generation and characterization

Ref. No.	Title	Summary	Related subjects
[4]	Cell type of origin influences the molecular and functional properties of mouse induced pluripotent stem cells	iPS cells from mouse fibroblasts, hematopoietic and myogenic cells exhibit distinct transcriptional and epigenetic patterns. Cellular origin influences the in vitro differentiation potentials of iPS cells	Tissue origin and differentiation potential
[5]	Induced pluripotent stem cells and embryonic stem cells are distinguished by gene expression signatures	Genome-wide data suggested that the iPSC signature gene expression differences are due to differential promoter binding by the reprogramming factors. Epigenetic memory of the donor tissue could be reset by serial reprogramming	Epigenetic memory
[6]	Epigenetic memory and preferential lineage-specific differentiation in induced pluripotent stem cells derived from human pancreatic islet beta cells	The pancreatic islet beta cell-derived iPS cells maintained open chromatin structure at key beta-cell genes, together with a unique DNA methylation signature. Those iPS cells demonstrated an increased ability to differentiate into insulin-producing cells compared with ES cells	Tissue origin and differentiation potential
[7]	Incomplete DNA methylation underlies a transcriptional memory of somatic cells in human iPS cells	A systematic comparison of iPS cells generated from hepatocytes, skin fibroblasts and melanocytes showed that iPS cells retain transcriptional memory of the original cells. The persistent expression of somatic genes can be partially explained by incomplete promoter DNA methylation	Incomplete promoter DNA methylation
[8]	Epigenetic memory in induced pluripotent stem cells	IPS cells harbor residual DNA methylation signatures characteristic of their somatic tissue of origin, which favors their differentiation along lineages related to the donor cell. The differentiation and methylation of nuclear transfer-derived pluripotent stem cells were more similar to ES cells	Epigenetic memory
[9]	Donor cell type can influence the epigenome and differentiation potential of human induced pluripotent stem cells	As a consequence of both incomplete erasure of tissue-specific methylation and aberrant de novo methylation, umbilical cord blood- and neonatal keratinocyte-iPS cells were distinct in genome-wide DNA methylation profiles and differentiation potential, implying that iPS cells retain ‘epigenetic memory’ of their tissue of origin	Tissue origin and differentiation potential
[10]	Cancer-related epigenome changes associated with reprogramming to induced pluripotent stem cells	Cancer-related epigenetic abnormalities arise early during reprogramming and persist in iPS cell colonies. These include hundreds of abnormal gene silencing events, patterns of aberrant responses to epigenetic-modifying drugs resembling those for cancer cells	Epigenetic abnormalities
[11]	Immunogenicity of induced pluripotent stem cells	In contrast to ES cells, abnormal gene expression in some cells differentiated from iPS cells can induce T cell-dependent immune responses in syngeneic recipients	Immune responses
[13]	Direct cell reprogramming is a stochastic process amenable to acceleration	The number of cell divisions is a key parameter driving epigenetic reprogramming to pluripotency. Almost all mouse donor cells are theoretically eventually give rise to iPS cells with continued growth and transcription factor expression	Stochastic model
[21]	DNA methylation dynamics in human induced pluripotent stem cells over time	Stochastic de novo methylation of genomic DNA occurs in iPS cell generation. Cell division proceeds in iPS cells after prolonged culture lead to a cell condition that epigenetically more closely resembles that observed in ES cells	Stochastic model
[67]	Enhanced generation of induced pluripotent stem cells from a subpopulation of human fibroblasts	Fibroblasts that expressed SSEA-3 demonstrated an enhanced iPS cell generation efficiency (~eightfold increase), while no iPSC derivation was obtained from the fibroblasts that did not express SSEA-3	Elite model
[30]	Multilineage-differentiating stress-enduring (Muse) cells are a primary source of induced pluripotent stem cells in human fibroblasts	Muse cells that are aleady pluripotent but are non-tumorigenic preexist in mesenchymal cells. In human fibroblasts, iPS cells are generated exclusively from Muse cells but never from non-Muse cells, suggesting that preexisting adult stem cells that are pluripotent selectively become iPS cells, but the remaining cells make no contribution	Elite model

As it now stands, the therapeutic use of iPS cells in patients is severely limited by the fact that iPS cells are immortal with the ability to cause tumors. Even if iPS cell-derived cells undergoing differentiation have a low risk of tumorigenesis, there are currently no realistic methods for resolving the issue of tumorigenesis. Thus, it is too difficult to detect and eliminate all the undifferentiated tumorigenic cells among the large number of iPS cells before therapeutic applications. In addition, the potential dangers posed by the uncontrolled and unstable genomes of iPS cells have been recently demonstrated by the analysis of several lines of ES and iPS cells [83].

Together, these issues reveal the strong need for a basic understanding of the iPS cell-generation mechanism. At any rate, the questions of what are iPS cells and how are they generated remain crucial issues to be resolved, and understanding the basic characteristics of iPS cells will advance the studies of these cells and their application.
